# Development of cancer metabolism as a therapeutic target: new pathways, patient studies, stratification and combination therapy

**DOI:** 10.1038/s41416-019-0666-4

**Published:** 2019-12-10

**Authors:** Adrian L. Harris

**Affiliations:** Molecular Oncology Laboratories, Weatherall Institute of Molecular Medicine, University of Oxford, Oxford, UK

## Abstract

Cancer metabolism has undergone a resurgence in the last decade, 70 years after Warburg described aerobic glycolysis as a feature of cancer cells. A wide range of techniques have elucidated the complexity and heterogeneity in preclinical models and clinical studies. What emerges are the large differences between tissues, tumour types and intratumour heterogeneity. However, synergies with inhibition of metabolic pathways have been found for many drugs and therapeutic approaches, and a critical role of window studies and translational trial design is key to success.

## Background

In this two-issue special edition on Cancer Metabolism in the *British Journal of Cancer*, which has been co-edited by me and Dr Christian Frezza, we consider in this first instalment the major clinical developments and trial designs with encouraging leads and mechanisms, which may translate to the clinic in the relatively near future. The second instalment includes more basic discoveries, new aspects and pathways, which need further investigation and development for therapy.

## Clinical trials

Several pathways have been targeted, often with many companies producing molecules against the same target, for example, lipid metabolism enzymes and tryptophan metabolism, but the first generation of compounds have been disappointing. However, the biochemistry on which they are based is sound, with extensive demonstration of upregulation of pathways in cell-line models, mouse models and in human cancers. For example, reprogramming of fatty acid metabolism in cancer has been well documented, and many different tumour types have upregulated fatty acid content, increased synthesis of fatty acids and their desaturation. The major role of phosphoinositide 3-kinase (PI3K), AKT and mammalian target of rapamycin (mTOR) in growth is well known, but they are also major modifiers of metabolism. The latter may contribute both to toxicity and therapeutic effect.

The reciprocal effects of metabolism on signalling and signalling on lipid metabolism are reviewed in the article by Koundouros and Poulogiannis^1^ on the mechanisms by which the lipids associated with tumour progression, including epigenetic modification and membrane fluidity. Fatty acid synthase (FASN) inhibitors have been developed, drugs such as TVB316, TVB2640 have been effective with less toxicity than predecessors and TVB2640 is now in trial. Lipid metabolism targets are also being developed as therapeutic strategies, for example, targeting stearoyl-CoA desaturase (SCD) enzymes, which produce monounsaturated fatty acids.

The discovery that immune cells, such as CD8 in contrast to regulatory T cells, are sensitive to tryptophan depletion and metabolites from tryptophan led to major efforts to inhibit the main enzymes involved in this pathway (reviewed by Opitz et al.^2^). Although nearly a dozen different compounds were developed and many tested in the clinic, the overall results have been disappointing. Opitz et al.^2^ describe in detail the trials, potential resistance mechanisms and the next-generation approaches to overcoming this problem.

Trial design is critical at the early steps of new drug development and needs to include a range of pharmacodynamic markers, particularly proof of the pathways inhibited in the tumour and that this has a biological effect. These studies are complex and often involve many different groups to maximally utilise the information available and ensure the right processing of biopsies and studies undertaken with appropriate approvals, and these are reviewed extensively in the paper by Aroldi and Lord.^3^

## Biomarkers

Proof of the presence of the target and its inhibition, plus the biological effect, is key to success with antimetabolite therapy. Essentially, this is patient stratification so that in future studies the potential benefits and effectiveness can be predicted. This has been a great challenge so far, but approaches to investigate the effects of metformin on the endometrium in patients with endometrial cancer are described in the paper by Crosbie and colleagues.^4^ A series of patients were treated with metformin, and detailed evaluation in tumour and cell lines showed microenvironmental influences, such as hypoxia and high glucose (common in diabetic patients who have a higher risk of endometrial cancer), mediate resistance.

In mesothelioma, as described by Urso et al.,^5^ genetic changes such as loss-of-function mutations in BRCA-associated protein 1 (BAP1), are important in metabolic re-wiring and provide a metabolic vulnerability. Another subset of cases show loss of methyladenosine phosphorylase (MTAP). These genetic changes provide routes for patient stratification and specific drug therapies.

Immunohistochemistry is one of the most widely available approaches to study cancer in the clinic and provide stratification. A large study in primary breast cancer by Green et al.^6^ showed that there was a strong association between amino acid transporters and upregulation of programmed death ligand 1 (PD-L1) and regulatory T cells. The expression was also associated with poor outcome, further evidence that metabolic pathways may be related to specific subtypes of immune infiltrates. Again, it helps to understand how drugs may be used together in the future targeting different components, for example, blocking PD-L1 and glutamine metabolism.

## Immune system

Other aspects of the immune system are reviewed, particularly myeloid-derived suppressor cells (MDSCs), which are a heterogenous population of cells and have multiple mechanisms producing poor prognosis on cancer. The role of amino acids, fatty acids and glucose in their function is reviewed by Yi and colleagues,^7^ and highlights how these cells could be targeted through metabolic inhibition. It needs to be remembered that any inhibition of metabolism will also affect stromal cells as well as normal tissues, and there may be further effects that could make antimetabolites effective, for example, in endothelial metabolism and fibroblasts.

## Combination therapies

Many drugs affect metabolic pathways and a switch to mitochondrial metabolism is being increasingly reported in several resistant cancers. In addition, because of the key effect growth factor receptor signalling has on metabolism, resistance to inhibition can be mediated via changes in metabolic pathways. Thus, the combination of a drug or therapy with antimetabolites provides a promising approach, both for selectivity and therapeutic effectivity.

In a clinical study of head and neck cancer by Zhang et al.,^8^ high levels of expression of amino acid transporter (ASCT2) and large neutral amino acid transporter 1 (LAT1) were found, and were associated with poor prognosis. The role of ASCT2 on glutamine uptake was analysed, and similarly the additional role of sodium-coupled neutral amino acid transporter 2 (SNAT2). Blocking amino acid uptake enhanced the effects of epithelial growth factor (EGF) receptor inhibition. This will require further preclinical study.

In melanoma cell lines resistant to the BRAF mutant inhibitor, vemurafenib, Delgado-Goñi et al.^9^ reported that there was a shift to mitochondrial metabolism and inflammatory lipid metabolism. There was heterogeneity in the mechanisms of resistance in vitro, which emphasises the complexity of inhibiting metabolism uniformly in a tumour. Genes involved in prostaglandin synthesis were amplified in ~5% of patients, who had a decreased survival, supporting a potential clinical relevance. Combining vemurafenib with emerging inhibitors of the enzymes may be a way to prevent the development of resistance.

Viruses alter the host metabolism to allow their reproduction and expansion, and this is true for oncolytic viro-immunotherapy. In hepatocellular carcinoma-bearing mice, Wei and colleagues^10^ described that the oncolytic Newcastle disease virus had antitumour effects, but in combination with the drug that blocked pyruvate dehydrogenase kinase (DCA, dichloroacetate), there was a much greater effect on the antitumour immune responses. This supports the concept that the metabolic environment within cancers has many immunosuppressive components, for example, lactate, decreased glucose availability, and as mentioned in other papers, tryptophan depletion. The effects were clear and showed promise for future investigations of the interactions of these pathways.

## Novel pathways

Human pancreatic cancers have been shown to upregulate protein kinase D1 (PKD1), a serine/threonine kinase, and Kumari et al.^11^ have investigated the underlying signalling mechanisms. Normal pancreatic tissues and chronic pancreatitis tissues showed very little expression, PKD1 expression was upregulated early in preneoplastic lesions and tumours and a new role was demonstrated in drug resistance.

Not all metabolic changes have to be increased to have a major effect on tumour biology. For example, it is shown in the study by Li et al.^12^ that low expression of 3-hydroxybutyrate dehydrogenase type 2 (BDH2) has a major effect on tumour iron metabolism in nasopharyngeal carcinoma. Apart from its effect on ketone body metabolism, BDH2 also affects intracellular iron concentrations, responds to iron and helps in the synthesis of a siderophore. BDH2 deficiency and knockdown have previously been shown to promote iron overload. Upregulation of BDH2 slowed down cell growth in vivo and in vitro. The mechanism was related to iron retention, which is much greater in the absence of BDH2 than in its presence. This suggests that low BDH2 expression may be a suitable marker for selecting patients for iron-chelation-targeted therapies.

## Conclusions

Cancer metabolism is a vibrant field with several clinical trials completed, many new pathways being discovered and the possibility of synergy with existing treatments being explored. In addition, a series of biomarkers and metabolic pathways that are up- or down-regulated may help in selectivity, but it is clear that patient selection is of the utmost importance, and analysing the relevant compartment for expression or suppression of the target mechanism is critical. Such studies are often best done with a window design. Overall, therefore, although the only marketed drug for a metabolic pathway in cancer (besides nucleotide metabolism) is for inhibition of a mutant enzyme IDH, there remains great scope for selective development of inhibitors for specific tumour types, but the heterogeneity of each type of cancer and even within the tumour needs to be taken into account. There is also likely to be significant interaction with hypoxia biology, which regulates many of these processes.

## Data Availability

Not applicable.

## References

[1] Koundouros, N. & Poulogiannis, G. Reprogramming of fatty acid metabolism in cancer. *Br. J. Cancer*10.1038/s41416-019-0650-Z (2019). 10.1038/s41416-019-0650-z PMC6964678 31819192

[2] Opitz, C. A., Somarribas Patterson, L. F., Mohapatra, S. R., Dewi, D. L., Sadik, A., Platten, M. & Trump, S. The therapeutic potential of targeting tryptophan catabolism in cancer. *Br. J. Cancer*10.1038/s41416-019-0664-6 (2019). 10.1038/s41416-019-0664-6 PMC6964670 31819194

[3] Aroldi, F. & Lord, S. R. Window of opportunity clinical trial designs to study cancer metabolism. *Br. J. Cancer*10.1038/s41416-019-0621-4 (2019). 10.1038/s41416-019-0621-4 PMC6964681 31819180

[4] Sivalingam, V. N., Latif, A., Kitson, S., McVey, R., Finegan, K. G., Marshall, K. et al. Hypoxia and hyperglycaemia determine why some endometrial tumours fail to respond to metformin. *Br. J. Cancer*10.1038/s41416-019-0627-y (2019). 10.1038/s41416-019-0627-y PMC6964676 31819173

[5] Urso, L., Cavallari, I., Sharova, E., Ciccarese, F., Pasello, G. & Ciminale, V. Metabolic rewiring and REDOX alterations in malignant pleural mesothelioma. *Br. J. Cancer*10.1038/s41416-019-0661-9 (2019). 10.1038/s41416-019-0661-9 PMC6964675 31819191

[6] El Ansaria, R., Crazea, M. L., Althobitia, M., Alfarsia, L., Ellisa, I. O., Rakhaa, E. A. & Green, A. R. Enhanced glutamine uptake influence composition of immune cells infiltrates in breast cancer. *Br. J. Cancer*10.1038/s41416-019-0626-z (2019). 10.1038/s41416-019-0626-z PMC6964696 31819174

[7] Hu, C., Pang, B., Lin, G., Zhen, Y. & Yi, H. Energy metabolism manipulates the fate and function of tumour myeloid-derived suppressor cells. *Br. J. Cancer*10.1038/s41416-019-0644-x (2019). 10.1038/s41416-019-0644-x PMC6964679 31819182

[8] Zhang, Z., Liu, R., Shuai, Y., Huang, Y., Jin, R., Wang, X. & Luo, J. ASCT2 (SLC1A5)-dependent glutamine uptake is involved in the progression of head and neck squamous cell carcinoma. *Br. J. Cancer*10.1038/s41416-019-0637-9 (2019). 10.1038/s41416-019-0637-9 PMC6964701 31819178

[9] Delgado-Goñi, T., Galobart, T. C., Wantuch, S., Normantaite, D., Leach, M. O., Whittaker, S. R. & Beloueche-Babari, M. Increased inflammatory lipid metabolism and anaplerotic mitochondrial activation follow acquired resistance to vemurafenib in BRAF-mutant melanoma cells. *Br. J. Cancer*10.1038/s41416-019-0628-x (2019). 10.1038/s41416-019-0628-x PMC6964672 31819183

[10] Meng, G., Li, B., Chen, A., Zheng, M., Xu, T., Zhang, H. et al. Targeting aerobic glycolysis by dichloroacetate improves Newcastle disease virus-mediated viro-immunotherapy in hepatocellular carcinoma. *Br. J. Cancer*10.1038/s41416-019-0639-7 (2019). 10.1038/s41416-019-0639-7 PMC6964686 31819179

[11] Kumari, S., Khan, S., Sekhri, R., Mandil, H., Behrman, S., Yallapu, M. M. et al. Protein kinase D1 regulates metabolic switch in pancreatic cancer via modulation of mTORC1. *Br. J. Cancer*10.1038/s41416-019-0629-9 (2019). 10.1038/s41416-019-0629-9 PMC6964700 31819177

[12] Li, B., Liao, Z., Mo, Y., Zhao, W., Zhou, X., Xiao, X. et al. Inactivation of 3-hydroxybutyrate dehydrogenase type 2 promotes proliferation and metastasis of nasopharyngeal carcinoma by iron retention. *Br. J. Cancer*10.1038/s41416-019-0638-8 (2019). 10.1038/s41416-019-0638-8 PMC6964698 31819181

